# Self-Assembly of Pyridine-Modified Lipoic Acid Derivatives on Gold and Their Interaction with Thyroxine (T4)

**DOI:** 10.3390/ijms14023500

**Published:** 2013-02-06

**Authors:** Willem M. Albers, Roberto Milani, Kirsi Tappura, Tony Munter, Giuseppe Resnati, Pierangelo Metrangolo

**Affiliations:** 1 VTT Technical Research Centre of Finland, Sinitaival 6, 33720 Tampere, Finland & Metallimiehenkuja 8, Espoo, 02044 VTT, Finland; E-Mails: kirsi.tappura@vtt.fi (K.T.); tony.munter@vtt.fi (T.M.); pierangelo.metrangolo@polimi.it (P.M.); 2 NFMLab-DCMIC “Giulio Natta”, Politecnico di Milano, Via Mancinelli 7, I-20131 Milano, Italy; E-Mail: giuseppe.resnati@polimi.it; 3 Center for Nano Science and Technology@PoliMi, Istituto Italiano di Tecnologia, Via Giovanni Pascoli, 70/3, I-20133 Milano, Italy

**Keywords:** thyroxine, self-assembly, imprinting, noncovalent interactions, Surface Plasmon Resonance (SPR)

## Abstract

Pyridyl derivatives of lipoic acid were prepared as ligands for the study of the interaction with thyroxine (**T4**). Thin self-assembled films of the ligands were prepared in 70% ethanol on gold and their interaction with **T4** was studied by titration experiments in an aqueous buffer solution using Surface Plasmon Resonance (SPR). The thickness and refractive index of the ligand layers were calculated from SPR spectra recorded in two media, also allowing for surface coverage and the density of the layers to be estimated. Two ligands, a 4-pyridyl and a bis(2-hydroxyethyl) derivative of lipoic acid, were selected to investigate the feasibility for producing molecularly imprinted self-assembled layers on gold for **T4**. The methodology was to co-assemble **T4** and the ligand onto the gold surface, elute the **T4** from the layer under alkaline conditions, and study the rebinding of **T4** to the layer. Multiple elution/rebinding cycles were conducted in different buffer solutions, and rebinding of T4 could be observed, with a moderate binding affinity that depended greatly on the solvent used. More optimal binding was observed in HBS buffer, and the affinity of the interaction could be slightly increased when the 4-pyridyl and bis(2-hydroxy-ethyl) derivatives of lipoic acid were combined in the imprinted layer.

## 1. Introduction

**Figure 1:**
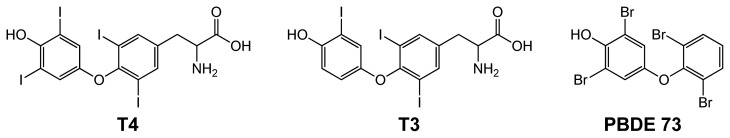
Molecular structures of **T4** (left), **T3** (mid), and the poly-brominated diphenyl ether **PBDE 73** (right).

Thyroxine (or 3,5,3′,5′-tetraiodo-l-thyronine, **T4**) and triiodothyronine (or 3,5,3′-triiodo-l-thyronine, **T3**) (Figure 1) are the two major thyroid hormones related to thyroid action, which is essential for the differentiation and growth of nearly all tissues, while also having marked influence on oxygen consumption, metabolic rate and RNA synthesis [1]. Their synthesis, secretion and transport are coordinated by the hypothalamus, pituitary and thyroid glands, and comprise various receptors, transport proteins and additional hormones. Thyrotropin Releasing Hormone (TRH) and Thyroid Stimulating Hormone (TSH) are the main regulators of the biosynthesis of **T4** and **T3** by a negative feedback mechanism. **T4** is synthesized in the thyroid gland by iodination and further covalent attachment of the phenyl rings from the amino acid tyrosine, while **T3**, the more potent hormone, is produced from **T4** by deiodination outside the thyroid gland [2].

Thyroid hormones are known to bind to various receptors, of which many crystallographic structures have already been elucidated [1]. Since these compounds are very hydrophobic, they are generally bound with high affinity into a hydrophobic pocket of the thyroid receptors [3,4], and their concentrations in blood serum are notoriously low (1.5–20 pM) [5]. Various carrier proteins are capable of solubilizing hydrophobic compounds, including a large collection of common drugs and vitamins like retinol. The main transporters of thyroid hormones in the blood stream are Thyroxine Binding Globulin (TBG), albumin, and transthyretin (pre-albumin) [3,6,7]. In particular, the binding of **T4** to transthyretin seems to involve not only hydrogen bonds, but also short I···O contacts [8], which can be identified as genuine halogen bonds, namely, noncovalent interactions involving halogen atoms as electrophilic species [9].

The attention to the biological relevance of **T4**-analogue halocarbons is strongly growing. It has been demonstrated that poly-brominated diphenyl ethers (PBDEs) show a remarkable thyroid hormonal activity as endocrine disrupting compounds (EDCs) [10]. This is not surprising when taking into account the structural similarities of PBDEs with **T4** (see Figure 1); however, the exact mechanism of interference with the thyroid hormonal action is not yet fully understood. The detection of **T4** and its analogues is therefore an important societal issue. Nowadays, in clinical practice, free **T4** and its mono-dehalogenated analogue **T3** are measured by various highly sensitive methods, such as radioimmunoassay (RIA), fluoroimmunoassay (FIA and TR-FIA) [11] or chemiluminescent assay. Recently, EDCs have been assayed with biosensor systems, e.g., utilizing thyroid transport proteins as receptors for EDCs [12,13].

Biosensor formats for **T4** generally make use of its natural receptors [12], but invariably need some form of signal amplification or competitive assay format, thus not complying always with the biosensor requirements [14]. With Surface Plasmon Resonance (SPR), the binding of **T4** and its tri-iodinated analogue **T3** can be studied in real time without the need for labeling [15].

Molecular imprinting is presently a broad field of research holding very high prospects for the detection of molecular targets, particularly drug substances [16,17]. The method is based on the templating effect of target molecules in a solidifying matrix (e.g., by polymerizing, freezing, or adsorbing/packing), as conducted in a suitable medium. The template is then removed from the matrix, expectedly leaving binding pockets selective for the target molecule. Imprinting is mostly achieved with cross-linked polymers as a matrix, but a viable alternative is represented by using the imprinted self-assembled monolayers (*i*-SAMs), *i.e.*, employing non-covalent interactions to assemble a surface layer in the presence of the templating molecule. Although the detection of small molecules with SPR may be cumbersome, a successful imprinting study was recently published on morphine [18].

**Figure 2:**
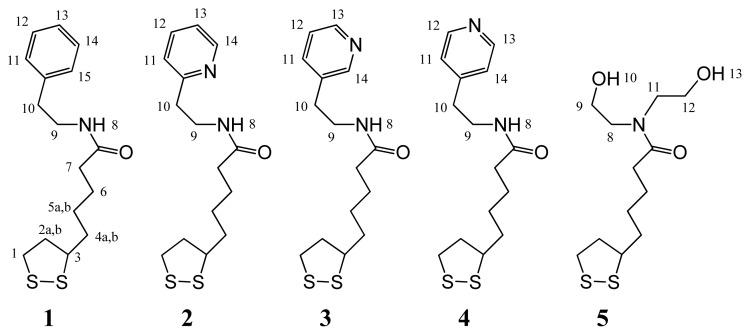
Lipoic acid derivatives used as ligands in the present work. Proton positions are labeled for NMR assignments.

The present work constitutes a preliminary study on the SPR detection of **T4** by depositing self-assembled, **T4**-imprinted thin layers on gold surfaces. In order to achieve this goal, we designed a series of pyridine-modified derivatives of lipoic acid (see Figure 2). All the ligands are expected to establish hydrogen and halogen bonds with **T4** in different measures. Their amide (**1**–**5**), pyridyl (**2**–**4**) and hydroxyl (**5**) groups are, in fact, capable of hydrogen bonding with the phenolic hydroxyl, amine, and carboxyl moieties of **T4**. The pyridyl derivatives **2**–**4**, in particular, were designed to also enable short contacts with the iodine atoms of **T4**. Halogen bonding based on iodoarene-pyridyl couples has been reported to have a binding strength comparable to that of phenol-pyridine hydrogen bonds [19], and generally higher than that of iodine-oxygen interactions such as those inferred from TTR-thyroxine structures [8]. In principle, a weak halogen bond involving the amidic oxygen is also possible. The control compound **1** lacks the pyridyl moiety, but is more likely to engage in hydrophobic interactions, indicated by its higher *logP* value (see Experimental Section). Compound **5** is a ligand that was previously studied in *i*-SAM formation for morphine [18], and was employed here for comparison purposes.

## 2. Results and Discussion

### 2.1. Self-Assembly of the Lipoates and Thin Layer Formation

**Figure 3:**
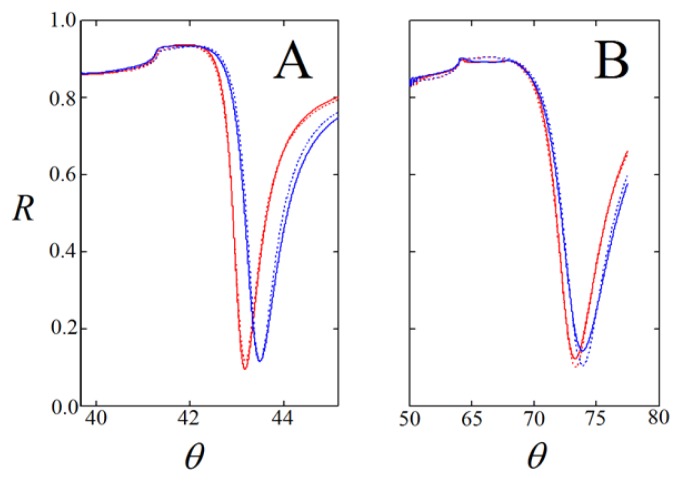
SPR curves of incident light angle (θ) *versus* reflection coefficient (*R*), measured in (**A**) air and (**B**) 70% ethanol. Red curves were obtained on blank gold, while blue curves were acquired after layer formation of lipoate **4**. Fitted data are represented by the dotted curves.

A large set of SPR data was acquired to obtain information about the thickness of the layers assembled from compounds **1**–**4**. The layers were prepared from 2 to 3 mM solutions of the ligands in 70% ethanol, according to the earlier work [18]. In particular, the two-medium method appeared to give useful estimates of the thickness and refractive index of the thin layers [20]. This method comprises the measurement of SPR shifts in two media (in this case air and 70% ethanol) and fitting of the SPR curves to theory in order to give an estimate for the thickness (*d*) and refractive index (*n*) of the layer. As an example, fitted SPR curves are given in Figure 3 for one of the measurements on ligand **4** as obtained with the SPR-Navi 200 spectrometer (Bionavis Ltd, Tampere, Finland). The angular shifts in air and 70% ethanol for all the compounds are given in Figure S1 and an example of the fitting contour plots for *n* and *d* are given in Figure S2 of the supporting information.

Table 1 summarizes the results for the thickness and refractive index (with standard error limits) of the deposited layers. Additionally, the density was calculated from the obtained refractive index using an estimated value of the molecular refractivity, while the surface coverage was subsequently calculated from the thickness and density values. These results indicated that the thickness of the layers was much more than a monolayer when the assembly was performed at 2–3 mM ligand concentration, particularly for compounds **2** and **3**. The density and the refractive index of the layers were slightly lower than the calculated values (see Experimental Section), thus indicating not very tightly packed layers, which is credible considering that the dithiolane ring needs more space on the surface than linear alkylmercaptans. The density of the lipoate layers appeared to be largely the same (1.0 g/cm^3^).

**T4** itself showed substantial layer formation onto the gold surface, and the observed refractive index was significantly smaller than the theoretical value (1.80). This might indicate that the layer includes solvent molecules. As can be reasonably expected, the density of the **T4** layer was higher (1.75 g/cm^3^) than that of the lipoates, but it was much lower than the theoretical value (2.64). Based on these results it can be postulated that **2** and **3** may have some tendency to dimerize by interaction between the pyridine and the amide hydrogen, and thus form thicker layers compared to **4**. The overall surface area per molecule of monolayers of the ligands was estimated by molecular calculations [22–24], and were approximately 37 Å^2^ per molecule for the pyridyl and phenyl ligands (**1**–**4**) and 48 Å^2^ per molecule for ligand **5**, yielding a surface coverage of 140 ng/cm^2^ and 100 ng/cm^2^, respectively. Thus, multilayers were formed at the conditions used, as indicated by the fractional surface coverage in Table 1.

### 2.2. Binding of **T4** to Self-Assembled Lipoate Layers

Initially, the binding of **T4** to the layers of ligands **1**–**4** on gold was studied with SPR using the Biacore 3000 instrument in phosphate buffered saline solution with 0.1% DMSO and pH = 9.0. High pH and DMSO were initially used to increase the solubility of **T4**. The self-assembly of ligands **1**–**4** was performed here *ex situ* before each binding experiment in 70% ethanol, which was a suitable solvent for all compounds used. Results were fitted to the Langmuir-Freundlich isotherm (see Experimental Section) to estimate the affinity constant (*K*_A_), the binding capacity (*Q*), and the binding exponent (ν).

**Figure 4:**
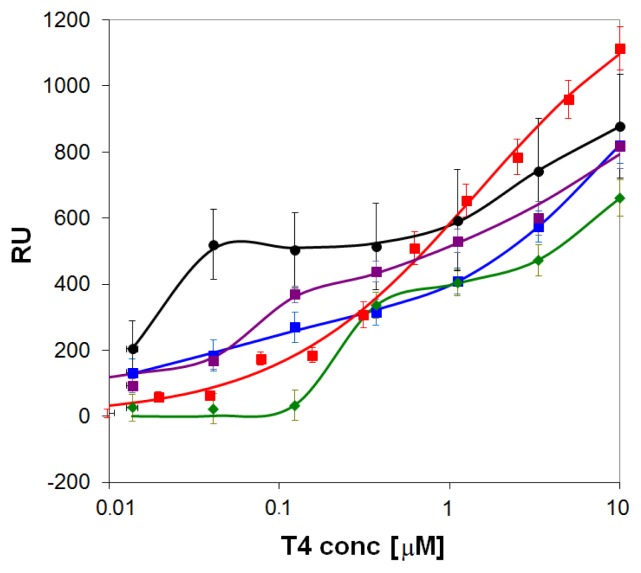
Adsorption isotherms of **T4** on clean gold ( 
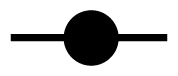
) and onto self-assembled layers of the pyridyl-lipoate conjugates **1** ( 
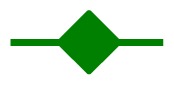
), **2** ( 
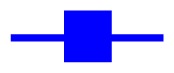
), **3** ( 
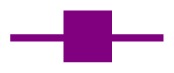
), and **4** ( 
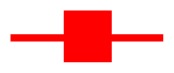
). Measurements were performed in PBS (150 mM NaCl, 50 mM phosphate buffer, pH = 9.0) supplemented with 0.1% DMSO.

The observed binding curves of **T4** were quite different for the various surfaces tested (Figure 4), and showed a marked step-wise response (with ν > 1) at a particular concentration for blank gold (*K*_A_ ≈ 7 × 10^7^) and compounds **1** (*K*_A_ ≈ 4 × 10^6^) and **3** (*K*_A_ ≈ 2 × 10^7^), while rather heterogeneous binding (ν < 1) was evidenced for the compounds **2** (*K*_A_ ≈ 3 × 10^7^) and **4** (*K*_A_ ≈ 7 × 10^5^). Although compound **4** had the lowest affinity, it showed the largest binding capacity for **T4** (*Q* = 1360 RU). Thus, rather characteristic differences were observed for the pyridyl-lipoate conjugates without imprinting. The **T4** adsorption level on the self-assembled layer of compound **1** (the control) was lowest: this appears to indicate that the expected higher tendency of ligand **1** to form hydrophobic interactions with **T4** cannot make up for the affinity loss caused by the lack of a pyridyl group capable of establishing hydrogen- and halogen bonds with **T4**. As compound **4** displayed the largest binding capacity, and has the pyridine nitrogen on a site that is more amenable to hydrogen and halogen bonding, it emerged as the most promising candidate for further study with imprinted layers. The strong affinity binding of **T4** for blank gold, however, should be taken in consideration as a possible obstacle to imprinting by self-assembly, because it may be difficult to elute the template from the layer.

### 2.3. Imprinted Self-Assembled Layers with **T4**

In order to achieve suitable conditions for layers formation that enable the observation of imprinting effects, preliminary experiments were performed with mixtures of **T4** as template molecule and compound **4** as ligand. The mixtures were also made in 70% ethanol and manually spotted (*ex situ*) on a peroxide-cleaned gold chip. The coated chip was mounted into the SPR instrument (Biacore 3000) and was subjected to an elution step, to remove **T4** from the layer (either with high or low pH buffer solutions). Hereafter, rebinding of **T4** was studied in aqueous buffer solutions (either PBS or HBS at pH = 7.4). The rebinding was performed at a single concentration of **T4**, or at a broad range of concentrations, constructing a titration curve.

**Figure 5:**
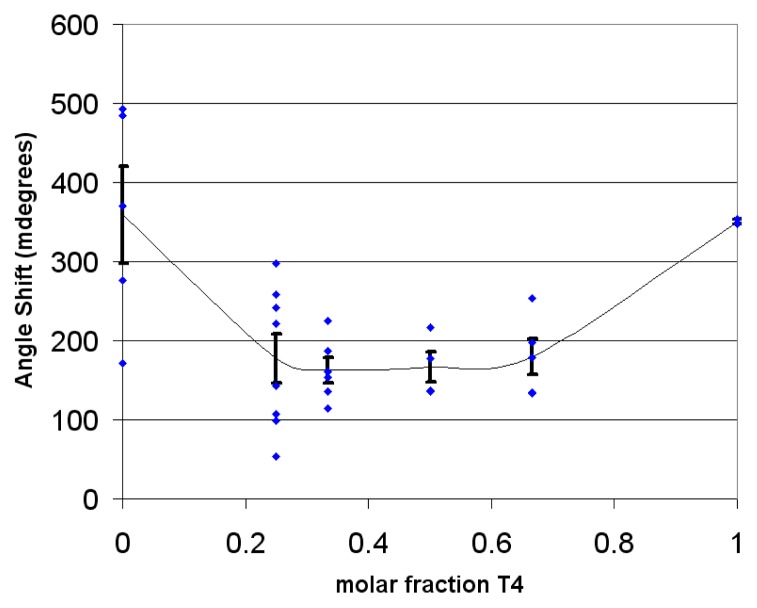
The SPR angle shift as a function of molar fraction of **T4** in a mixture with ligand **4**. The data points are given in blue, and the averages (with error bars) plotted in black. At molar fraction 0.0 the SPR shift is due to pure ligand **4**, while at fraction 1.0 the SPR shift is due to pure **T4**.

The solvent for layer formation (70% ethanol) was chosen for its ability to dissolve both **T4** and **4**. However, such solvent was damaging the fluidics system of the Biacore 3000 instrument, and thus, in some cases, *in situ* layer formation in this solvent was measured separately with another SPR instrument in 70% EtOH (the SPR-Navi 200) (a screen shot of an *in situ* measurement is given in the supporting information, Figure S3). It was observed that mixtures of **T4** and **4** gave a somewhat lower SPR response compared to that of the pure compounds, indicating formation of slightly thinner (or less dense) layers for the mixtures than for the pure compounds (Figure 5).

**Figure 6:**
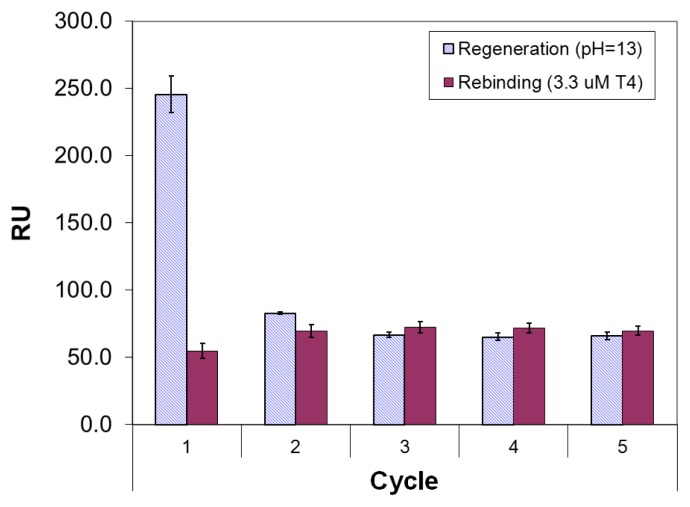
SPR response to five cycles of elution/rebinding of a binary layer of **T4** (1 mM) and **4** (2 mM), using HBS running buffer. The regeneration agent was 0.1 M NaOH and the **T4** standard was set at 3.3 μM in HBS buffer. The negative SPR shift of the regeneration step was plotted in positive direction to allow comparison with the rebinding of **T4**.

Since mixing of **T4** with **4** had a significant effect on the layer formation, the first tests with *ex situ* imprinted layers were made at various molar ratios of **4** with **T4**, also with reference to ligand **5** as studied in the earlier work [18]. In the various trials, the binding of **T4** to binary layers of **T4** and ligand **4** was studied initially in PBS buffer, using various regeneration reagents (see Figure S4). It was noticed that denser (or thicker) layers were produced with ligand **4** in comparison with ligand **5**, and that there was a maximum in layer thickness for the molar ratio (**T4**/**4**) of 1:2. However, PBS buffer gave rise to a decreasing trend in repeated elution/rebinding tests, while stable regeneration cycles could be attained with HBS buffer (pH = 6.8). This was evidenced on layers formed *ex situ* from 1.0 mM **T4** and 2.0 mM **4** in 70% ethanol, using 0.1 M NaOH as the regeneration reagent (Figure 6) in HBS buffer. Thus, elution and rebinding of **T4** on imprinted layers of **4** and **5** were studied further using similar conditions as in Figure 6, employing titrations of **T4** after each elution step. As far as regeneration conditions are concerned, treatment with strong alkaline solution appeared to be more effective than treatment with 1 M HCl. This can be easily explained by the enhanced solubility of **T4** at higher pH, when de-protonation of the phenolic hydroxyl occurs.

**Figure 7:**
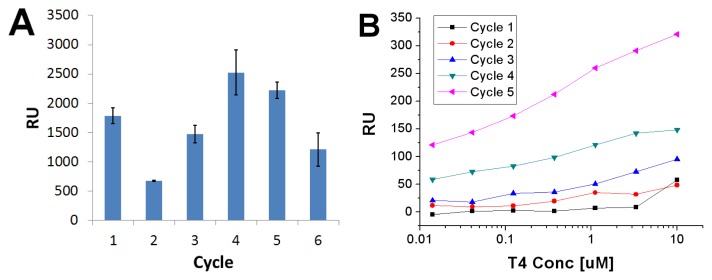
(**A**) Amount of complex desorbed upon successive elution steps with 0.1 M NaOH; (**B**) Rebinding of **T4** after elution. The running buffer was HBS.

When 2 mM **4** was mixed with 0.5 mM **T4** and spotted *ex situ* onto clean gold, a total film thickness of 11,700 (±170) RU was attained (in 70% EtOH), which represents a much thicker multilayer of **T4** and **4**. The first elution with 0.1 M NaOH caused a dissociation of 1790 (±140) RU, and subsequent elution steps after the titrations also resulted in rather substantial dissociation (Figure 7A). Titration with **T4** in the concentration range 0.014–10 M between these elution steps produced only minor binding initially, but upon repeated elution significant rebinding of **T4** started to occur (Figure 7B). The binding constants of the fifth cycle were: *K*_A_ = 1.4 × 10^6^ M^−1^, *Q* = 475 RU and ν = 0.29, indicating a low affinity heterogeneous rebinding process. The binding observed here was lower than the one found for **T4** on non-imprinted ligand **4** layers. Although these two binding experiments were performed in somewhat different conditions and are therefore not directly comparable, this may be an indication of a non-optimal efficiency of the regeneration of the imprinted layers, in which a significant amount of the binding sites would remain occupied by the **T4** molecules.

Similar experiments conducted with compound **5** and with a mixture of **4** and **5** yielded similar characteristics: an increasing response of the layer upon elution (see Figure S5). These results indicated that by using ligand **4** it was indeed possible to obtain rebinding, albeit at a relatively low affinity. For ligand **5** the binding constants obtained were *K*_A_ = 5.6 × 10^4^ M^−1^, *Q* = 678 RU and ν = 0.25, which signifies a lower affinity and higher degree of heterogeneity compared to ligand **4**. The lower affinity value may also be due to ligand **5** being more hydrophilic than **4** and therefore less prone towards hydrophobic interactions, although these already appeared to play a secondary role in the binding of **T4** to non-imprinted layers. Use of both ligands **4** and **5** in molar ratio 1:1 for the formation of an imprinted layer yielded the following binding constant: *K*_A_ = 2.7 × 10^6^ M^−1^, *Q* = 220 RU and ν = 0.39. Thus, a minor synergistic effect of the two ligands could be discerned, producing a higher affinity and slightly lower heterogeneity compared to the use of ligand **4** alone.

## 3. Experimental Section

### 3.1. Materials and Methods

For preparation of the ligands, lipoic acid, phenethylamine, 2-(2-aminoethyl)-pyridine, 4-(2-aminoethyl)-pyridine or 3-(2-aminoethyl)pyridine hydrobromide, and hydroxysuccinimide were obtained from Fluka and Aldrich. The ethanol used in this study was AA grade from Altia (Rajamäki, Finland). Other solvents used were DMSO (Baker). Buffer solutions were PBS (150 mM NaCl, 10 mM phosphate pH = 7.45) or HBS (150 mM NaCl, 10 mM HEPES pH = 6.8).

^1^HNMR spectra were recorded in CDCl_3_ on a Varian Oxford 300 instrument (300 MHz). Electro-spray mass spectrometry (ESI-MS) was performed on a LTQ Orbitrap XL spectrometer (Thermo Scientific, Waltham, MA, USA) in positive mode. For the characterization of compound 5 a different MS instrument was used: a Bruker MicrOTOF-Q in positive ion electrospray mode.

Binding studies in 70% ethanol were performed on a SPR-Navi 200 instrument (BioNavis Ltd., Ylöjärvi, Finland), which records whole SPR spectra (reflectivity *vs.* angle), while in aqueous environment the binding was predominantly studied with the Biacore 3000 instrument (Biacore Life Sciences, Uppsala, Sweden). These instruments have different measurement wavelengths (SPR-Navi at 670 nm, Biacore 3000 at 760 nm), and thus the results are not directly comparable. For the SPR-Navi the measured unit was in degrees or millidegrees of angular shift of the SPR minimum. The measured parameter for the Biacore 3000 was the “resonance unit” (RU). 1 RU represents a 10^−4^ degree angular shift of the SPR minimum, and for common biomolecules corresponds to a surface coverage of 1 pg/mm^2^ [25]. This estimate may be applied to the lipoate layers, which have similar refractive index as biomolecules, but not to **T4** due to its markedly higher molar refractivity.

### 3.2. Preparation of Lipoamide Derivatives

In a typical synthesis, lipoic acid (1.47 mmol) and *N*-hydroxy-succinimide (1.61 mmol) were dissolved in 10 mL of THF. Dicyclohexylcarbodiimide (1.75 mmol) was added, and precipitation occurred within few minutes. The mixture was stirred overnight at room temperature, and the solid precipitate was filtered off.

According to cases, phenethylamine, 2-(2-aminoethyl)pyridine, 4-(2-aminoethyl)-pyridine or 3-(2-aminoethyl)pyridine dihydrobromide (1.59 mmol) was added (note: for the hydrobromide salt, also 11.3 mmol sodium bicarbonate and 5 ml distilled water were added), and the mixture was stirred overnight at room temperature. The solvent was evaporated on a rotation evaporator, and addition of 15 mL dichloromethane allowed partial re-dissolution of the solid. The precipitate was filtered off. The solution was treated three times with 10 mL of saturated KCl aqueous solution, and the organic phase was dried on anhydrous sodium sulphate and filtered on paper.

Purification was performed by column chromatography using 5/1 or 10/1 (*v*/*v*) dichloromethane/ethanol mixtures as an eluent, according to cases. Pure products were recovered in about 55% yield for pyridyl derivatives, and in 70% yield for the phenyl derivative. Solubility of the ligands and thyroxine was generally low in aqueous solutions, but fairly good in 70% ethanol at the used concentrations for imprinting (10 mg/mL stock).

Characterization of the compounds was as follows. Theoretical values of the LogP (the octanol/water distribution coefficient, a measure of the hydrophobicity of the compound), molecular refractivity (*A*), refractive index (*n*) and density (ρ) were calculated with the ACD/Labs software [22–24].

#### *5-(1,2-Dithiolan-3-yl)-*N*-[(2-phenyl-ethyl)]pentanamide* (**1**, phenylethyl lipoamide, *M*_r_ = 309.49)

^1^HNMR (CDCl_3_): δ 7.34–7.18 (m, 5H; H_11_ to H_15_); 5.40 (br s, 1H; H_8_); 3.57–3.49 (m, 3H; H_3_, H_9_); 3.18–3.09 (m, 2H; H_1_); 2.82 (t, 2H, *^3^J_H,H_* = 7.2 Hz; H_10_); 2.45 (m, 1H; H_2a_); 2.12 (t, 2H, *^3^J_H,H_* = 6.9 Hz; H_7_); 1.90 (m, 1H; H_2b_), 1.64 (m, 4H; H_4a_, H_5a_, H_6_), 1.44 (m, 2H; H_4b_, H_5b_).MS (ESI) (M + H)^+^: found 310.12834. Physical properties: LogP = 3.57 ± 0.42; *R_m_* = 90.8 ± 0.3 cm^3^; *n* = 1.58 ± 0.02; ρ = 1.13 ± 0.06 g/cm^3^.

#### *5-(1,2-Dithiolan-3-yl)-*N*-[2-(pyridin-2-yl)ethyl]pentanamide* (**2**, 2-pyridylethyl lipoamide, *M*_r_ = 310.48)

^1^HNMR (CDCl_3_): δ 8.53 (d, 1H, *^3^J_H,H_* = 4.8 Hz; H_14_); 7.61 (t, 1H, *^3^J_H,H_* = 8.1 Hz; H_12_); 7.16 (d, 1H, *^3^J_H,H_* = 6.3 Hz; H_11_); 7.15 (t, 1H, *^3^J_H,H_* = 6.0 Hz; H_13_); 6.41 (br s, 1H; H_8_); 3.67 (dt, 2H, *^3^J_H,H_* = 6.0 Hz; H_9_); 3.54 (m, 1H; H_3_); 3.20-3.05 (m, 2H; H_1_); 2.98 (t, 2H, *^3^J_H,H_* = 6.6 Hz; H_10_); 2.43 (m, 1H; H_2a_); 2.15 (t, 2H, *^3^J_H,H_* = 7.5 Hz; H_7_); 1.89 (m, 1H; H_2b_); 1.66 (m,4H; H_4a_, H_5a_, H_6_); 1.45 (m, 2H; H_4b_, H_5b_). MS (ESI) (M + H)^+^: found 311.12325. Physical properties: LogP = 2.07 ± 0.43; *R_m_* =88.9 ± 0.3 cm^3^; *n* = 1.58 ± 0.02; ρ = 1.16 ± 0.06 g/cm^3^.

#### *5-(1,2-Dithiolan-3-yl)-*N*-[2-(pyridin-3-yl)ethyl]pentanamide* (**3**, 3-pyridylethyl lipoamide, *M*_r_ = 310.48)

^1^HNMR (CDCl_3_): ^1^HNMR (CDCl_3_): δ 8.47 (d, 1H, *^3^J_H,H_* = 4.5 Hz; H_13_); 8.44 (s, 1H; H_14_); 7.53 (d, 1H, *^3^J_H,H_* = 7.8 Hz; H_11_); 7.24 (dd, 1H, *^3^J_H,H_* = 4.8 and 7.5 Hz; H_12_); 5.57 (br s, 1H; H_8_); 3.60-3.49 (m, 3H; H_3_, H_9_); 3.21-3.06 (m, 2H; H_1_); 2.83 (t, 2H, *^3^J_H,H_* = 6.9 Hz; H_10_); 2.47 (m, 1H; H_2a_); 2.14 (t, 2H, *^3^J_H,H_* = 6.9 Hz; H_7_); 1.91 (m, 1H; H_2b_); 1.66 (m,4H; H_4a_, H_5a_, H_6_); 1.44 (m, 2H; H_4b_, H_5b_). MS (ESI) (M + H)^+^: found 311.12360. Physical properties: LogP = 2.07 ± 0.43; *R_m_* =88.9 ± 0.3 cm^3^; *n* = 1.58 ± 0.02; ρ = 1.16 ± 0.06 g/cm^3^.

#### *5-(1,2-Dithiolan-3-yl)-*N*-[2-(pyridin-4-yl)ethyl]pentanamide* (**4**, 4-pyridylethyl lipoamide, *M*_r_ = 310.48)

^1^HNMR (CDCl_3_): δ 8.53 (d, 2H; *^3^J_H,H_* = 4.5 Hz; H_12_, H_13_); 7.13 (d, 2H, *^3^J_H,H_* = 4.2 Hz; H_11_, H_14_); 5.42 (br s, 1H; H_8_); 3.60–3.49 (m, 3H; H_3_, H_9_); 3.22–3.07 (m, 2H; H_1_); 2.84 (t, 2H, *^3^J_H,H_* = 6.6 Hz; H_10_); 2.45 (m, 1H; H_2a_); 2.14 (t, 2H, *^3^J_H,H_* = 6.6 Hz; H_7_); 1.90 (m, 1H; H_2b_); 1.73–1.41 (m, 6H; H_4a,b_, H_5a,b_, H_6_). MS (ESI) (M + H)^+^: found 311.12329. Physical properties: LogP = 2.07 ± 0.43; *R_m_* = 88.9 ± 0.3 cm^3^; *n* = 1.58 ± 0.02; ρ = 1.13 ± 0.06 g/cm^3^.

#### *5-(1,2-Dithiolan-3-yl)-*N*,*N*-bis(2-hydroxy-ethyl)-pentanamide* (**5**, di-(2-hydroxyethylamino)-lipo-amide, *M*_r_ = 293.45)

^1^HNMR (CDCl_3_): δ 3.93 (br s, 2H, H_10_, H_13_); 3.81 (t, 2H, *^3^J_H,H_* = 4.8 Hz; H_9_ or H_12_); 3.76 (t, 2H, *^3^J_H,H_* = 4.7 Hz, H_9_ or H_12_); 3.57 (m, 1H, H_3_); 3.52 (t, 2H, *^3^J_H,H_* = 5.1 Hz; H_8_ or H_11_); 3.48 (t, 2H*,^3^J_H,H_* = 5.2 Hz; H_8_ or H_11_); 3.13 (m, 2H; H_1_); 2.45 (m, 1H; H_2a_); 2.40 (t, 2H, *^3^J_H,H_* = 7.5 Hz; H_7_); 1.90 (m, 1H; H_2b_); 1.66 (m, 4H; H_4a_, H_5a_, H_6_); 1.46 (m, 2H; H_4b_, H_5b_). MS (ESI) (M + H)^+^: found 294.1267. Physical properties: LogP = 1.07 ± 0.38; *R_m_* =78.7 ± 0.3 cm^3^; *n* = 1.57 ± 0.02; ρ = 1.220 ± 0.06.

### 3.3. Preparation of the Imprinted Films and Titration Experiments

The *ex situ* preparation of the imprinted films consisted of the following steps: (1) cleaning of a gold chip in boiling NH_4_OH (30%)/H_2_O_2_ (30%)/H_2_O (1:1:7 *v*/*v*); (2) rinse of the slide with fresh MilliQ-grade water and immersion in 70% ethanol; (3) blowing dry the chip with a burst of inert gas and dispensing of 50–150 μL of a mixture of template (**T4**) and ligand at specified molar ratio, allowing the self-assembly to take place in a small Petri dish for 1 hour in 70% ethanol; (4) washing the slide with 70% ethanol and drying in a laminar flow cabinet in air, and (5) gluing the gold substrate in a Biacore cassette and inserting it in the Biacore 3000 instrument; (6) recording a baseline in buffer solution for at least 0.5 h until a stable baseline was obtained. Hereafter, elution solution (0.1 M NaOH) was injected and the amount desorbed (in “resonance units”, RU) recorded, and series of standards of **T4** in buffer solution were injected to construct a titration curve. Various cycles were run to see if there was any change in the response as a result of regeneration. Titration curves were fitted to the Langmuir-Freundlich (or Sips) isotherm (Equation 1), in which C is the molar concentration of the adsorbing species, *R* the SPR response and *Q* is the binding capacity (maximum binding response, sometimes also denoted as *R*_max_), *K*_A_ the affinity constant, and **ν** the binding exponent. ν = 1 indicates normal Langmuir binding, ν < 1 indicates heterogeneity of binding sites, while ν > 1 indicates cooperativity of binding [26].

(1) $$R=\frac{Q{\left(K_{\text{A}}C\right)}^{v}}{1+{\left(K_{\text{A}}C\right)}^{v}}$$

The preparation of films onto gold was also studied in real time (*in situ*) with the SPR-Navi instrument by inserting a larger gold chip after peroxide cleaning into the instrument, and docking it to a 2-channel, temperature-controlled fluidic system as part of a typical FIA-configuration. A running solution of 70% ethanol was applied by use of a peristaltic pump at set at 20 μL/min. The mixture of template and ligand was injected twice via an injection valve into the solvent stream. The angular shift can be followed in real time, but also the whole SPR curves can be recorded. The refractive index and thickness of the films were calculated with a recently developed method, which utilizes SPR spectra recorded in two media of known refractive index before and after film deposition [27]. The fitting algorithm utilized a theoretical model of the SPR phenomenon based on the Müller matrix representation of polarized light reflected from stratified thin optical layers [20].

## 4. Conclusions

The results indicated that the three pyridyl derivatives of lipoic acid showed multilayer formation at concentrations comparable to those of an earlier study (1–2 mM) [18]. The self-assembled layers of compounds **1**–**4** interacted with **T4** layers in quite different fashion. The largest binding capacity was obtained for ligand **4** which showed an affinity constant of *K*_A_ ≈ 7 × 10^5^ M^−1^. When depositing mixed layers of **T4** and ligand **4** to assess the possibility of molecular imprinting, the best rebinding effects could be observed in HBS buffer (pH = 7.45), attaining an affinity constant of 1.4 × 10^6^ M^−1^. A combination of ligand **4** with ligand **5** slightly increased the affinity (*K*_A_ = 2.7 × 10^6^ M^−1^). However, up to five regeneration cycles were required to obtain any response to **T4**, using a strong 0.1 M NaOH elution reagent. This is likely due to the removal of excess, weakly bound species, while stronger ligand-template interactions took place at sites in closer vicinity of the gold surface.

These results should however be considered preliminary, as a definitive confirmation of the imprinting effect would require the binding experiments to be performed in the same conditions as the imprinted layer formation. This could not be achieved in the present work, as the pyridyl ligands required deposition in ethanol, while binding/rebinding studies with SPR could only be performed in aqueous buffer solutions. A solution to this problem could be either the synthesis of more water-soluble ligands, or performing both imprinting and binding studies in ethanol (or other suitable organic solvent). The latter would, however, require a chemically more robust SPR setup, with a fluidics unit that can stand organic solvent well without giving excessive drift in the SPR response. However, it will be evident that the first alternative is more attractive for practical applications.

Our results point out also the role of the pyridyl moiety in the formation of imprinted layers with moderate affinity, either through hydrogen bonding and/or through the establishment of halogen bonds with the iodine atoms of **T4**. The relative importance of these two interactions could be investigated by using **T4** analogues dehalogenated to different degrees, and will be the object of further studies.

## Figures and Tables

**Table 1:** Physicochemical constants of the ligands 1–5 and **T4** assembled on clean gold in 70% EtOH from a concentration of 2 mM (**T4**, 5) or 3 mM (1–4): Molecular weight (*M_r_*), molar refractivity (*A*), refractive index (*n*), layer thickness (*d*), density (ρ), surface coverage (Γ), and fractional surface coverage (Φ).

Compound	*M_r_*	*A*^a^	*n*^b^	*d* (nm)	ρ (g/cm^3^) c	Γ (ng/cm^2^)	Φ d
1	309.49	90.8	1.48 ±0.04	2.2 ±0.4	0.96 ±0.06	221 ±33	1.6
2	310.48	88.9	1.51 ±0.02	4.2 ±0.3	1.04 ±0.04	451 ±16	3.2
3	310.48	88.9	1.47 ±0.01	5.7 ±0.13	0.99 ±0.01	560 ±11	4.0
4	310.48	88.9	1.51 ±0.02	3.1 ±0.23	1.05 ±0.03	322 ±14	2.3
5	293.45	78.7	1.47 ±0.02	1.8 ±0.1	1.03 ±0.04	180 ±4	1.8
T4	776.87	123.0	1.47 ±0.02	1.9 ±0.4	1.75 ±0.11	320 ±95	--

a The molar refractivity *A* and logP values were estimated using software from ADC Labs [21];

b Standard error = σ/√n.

c Density of the layer was calculated from the values of *M_r_*, *R_m_*, and *n* according to the relation: ρ = (*n*^2^ − 1)/(*n*^2^ + 2)·*M_r_*/*A*.

d Fractional surface coverage is the amount of monolayers, based on the estimated surface coverage of a monolayer.

## Supplementary Files

Supplementary File 1 Supplementary Information (PDF, 343 KB)
