# Supplementary material for: Self-Assembly of Pyridine-Modified Lipoic Acid Derivatives on Gold and Their Interaction with Thyroxine (T4)
Source: Int J Mol Sci. 2013 Feb 6;14(2):3500–13. doi: 10.3390/ijms14023500 (PMC3588055; doi:10.3390/ijms14023500)
Supplement: Supplementary File 1 — Supplementary Information (PDF, 343 KB) [file ijms-14-03500-s001.pdf]

Supplementary Information

Figure S1. SPR shifts for all the ligands and **T4** measured in 70% ethanol and in air with the SPR-Navi instrument.

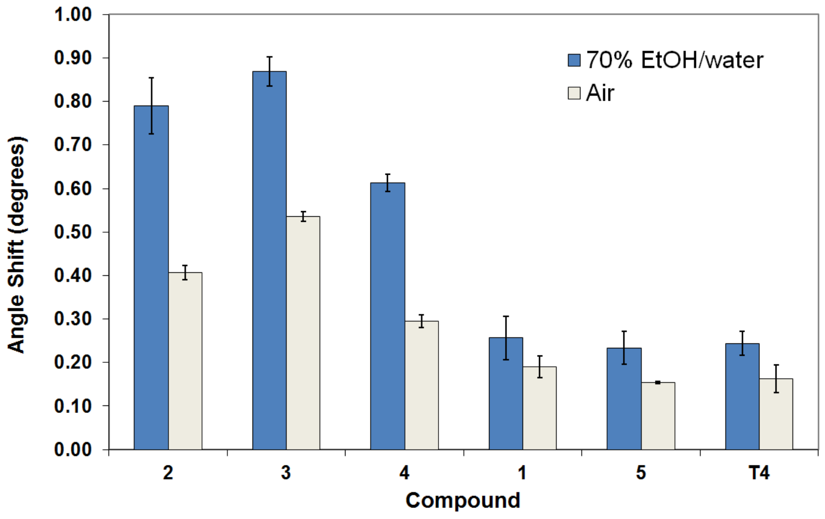

Figure S2. Goodness of fit $(1/\chi(n,d)^2)$ contour plots of n *vs.* d (**A**) in air (**B**) in 70% ethanol, and (**C**) combined $(1/[(1/\chi(n,d)^2_{air}) + (1/\chi(n,d)^2_{EtOH})])$. The parameters used were: λ = 670 nm, glass: n = 1.5315, air: n = 1.0005, 70% ethanol: n = 1.361, gold: d = 53.16 nm, n = 0.22, k = 3.909. The global solution of fitting for the self-assembled film was: n = 1.494, d = 3.403 nm.

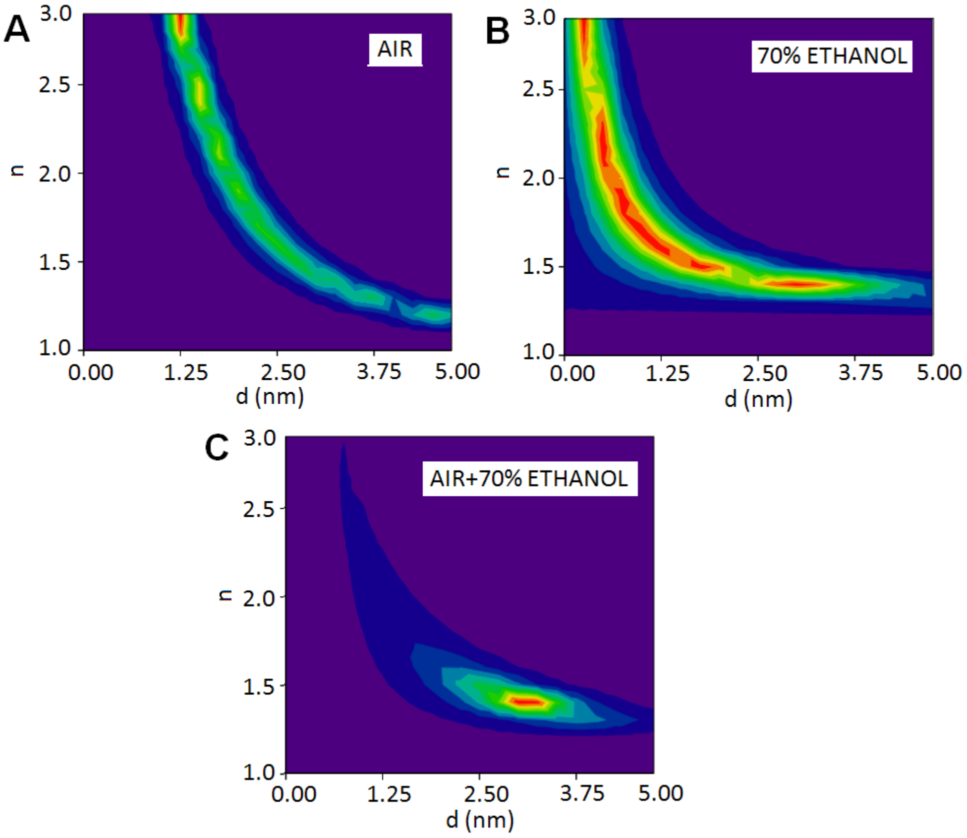

Figure S3. SPR shift of two injections of T4 and 4-Pyr-Lipa, mixed in the molar ratio 1:2 measured on the SPR-Navi instrument in 70% EtOH. A duplicate measurement of the same mixture was recorded in two flow channels (ch1 and ch2) simultaneously. Arrows indicated the injection time point. **Δ*R*** is the change in angle measured at the end of the injections (shown only for Channel 1).

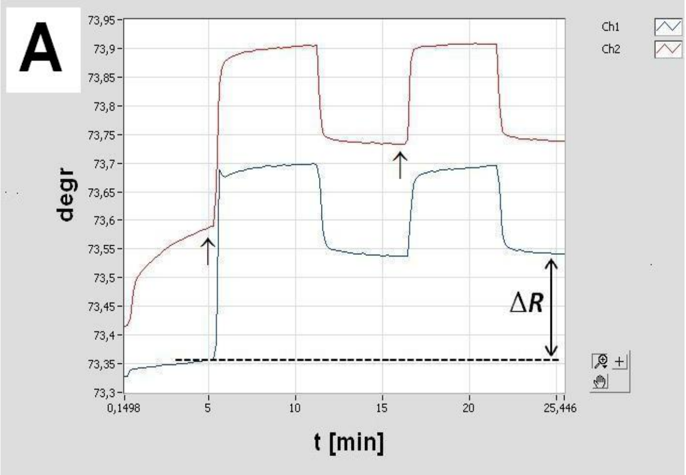

Figure S4. SPR shifts measured with the Biacore 3000 in PBS buffer upon **T4** binding (blue bars) and regeneration with high pH buffer (pH = 9.5, green bars) and low pH buffer (pH = 2.0, red bars). Results are shown for four surfaces prepared by self-assembly of **T4** with **4** and **5** in different molar ratios (e.g., 1:3 = 1 mM T4 and 3 mM ligand). The response of the regeneration solutions was always negative, but shown positive to enable comparison with the **T4**-binding.

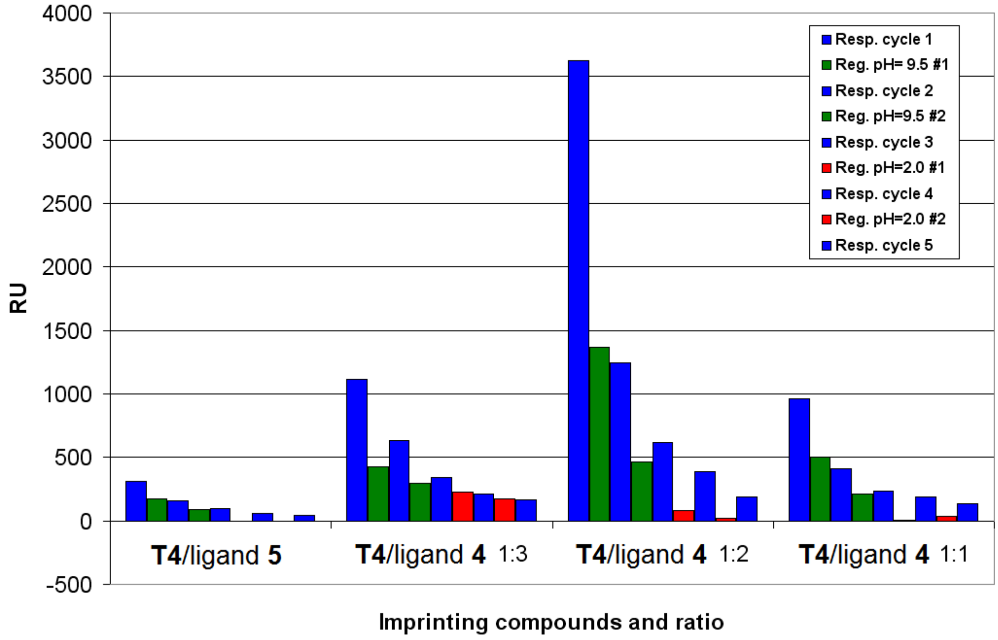

Figure S5. Titration curves for **T4** after sequential elution steps with 0.1 M NaOH of (**A**) A binary layer of compound **5** and **T4** deposited at concentrations of 2 mM and 0.5 mM respectively. (**B**) A ternary layer of compounds **4**, **5** and **T4** deposited at concentrations of 1 mM, 1 mM and 0.5 mM respectively. The depositions were performed by spotting on clean gold in 70% ethanol, and the binding curves were studied in HBS buffer with the Biacore 3000 instrument.

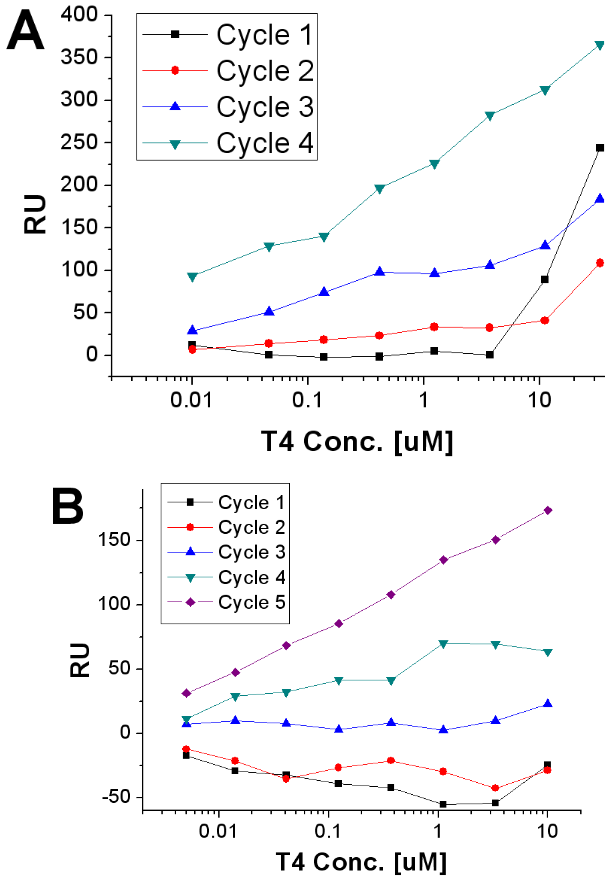

© 2013 by the authors; licensee MDPI, Basel, Switzerland. This article is an open access article distributed under the terms and conditions of the Creative Commons Attribution license (http://creativecommons.org/licenses/by/3.0/).
